# Clinical comparison of percutaneous transforaminal endoscopic discectomy and unilateral biportal endoscopic discectomy for single-level lumbar disc herniation

**DOI:** 10.3389/fsurg.2022.1107883

**Published:** 2023-01-16

**Authors:** Xiaokang Cheng, Beixi Bao, Yuxuan Wu, Yuanpei Cheng, Chunyang Xu, Yang Ye, Chentao Dou, Bin Chen, Hui Yan, Jiaguang Tang

**Affiliations:** ^1^ Department of Orthopedics, Beijing Tongren Hospital, Capital Medical University, Beijing, China; ^2^ Department of Orthopedics, Chengde Medical University Affiliated Hospital, Chengde, China; ^3^ Department of Orthopedics, China-Japan Union Hospital of Jilin University, Changchun, China

**Keywords:** lumbar disc herniation, percutaneous transforaminal endoscopic discectomy, unilateral biportal endoscopic discectomy, endoscopic, minimally invasive surgery

## Abstract

**Purpose:**

To compare the clinical outcomes of percutaneous transforaminal endoscopic discectomy (PTED) and unilateral biportal endoscopic discectomy (UBE) for the treatment of single-level lumbar disc herniation (LDH).

**Materials and methods:**

From January 2020 to November 2021, 62 patients with single-level LDH were retrospectively reviewed. All patients underwent spinal surgeries at the Affiliated Hospital of Chengde Medical University and Beijing Tongren Hospital, Capital Medical University. Among them, 30 patients were treated with UBE, and 32 were treated with PTED. The patients were followed up for at least one year. Patient demographics and perioperative outcomes were reviewed before and after surgery. The Oswestry Disability Index (ODI), visual analog scale (VAS) for back pain and leg pain, and modified MacNab criteria were used to evaluate the clinical outcomes. x-ray examinations were performed one year after surgery to assess the stability of the lumbar spine.

**Results:**

The mean ages in the UBE and PTED groups were 46.7 years and 48.0 years, respectively. Compared to the UBE group, the PTED group had better VAS scores for back pain at 1 and 7 days after surgery (3.06 ± 0.80 vs. 4.03 ± 0.81, *P* < 0.05; 2.81 ± 0.60 vs. 3.70 ± 0.79, *P* < 0.05). The UBE and PTED groups demonstrated significant improvements in the VAS score for leg pain and ODI score, and no significant differences were found between the groups at any time after the first month (*P* > 0.05). Although the good-to-excellent rate of the modified MacNab criteria in the UBE group was similar to that in the PTED group (86.7% vs. 87.5%, *P* > 0.05), PTED was advantageous in terms of the operation time, estimated blood loss, incision length, and length of postoperative hospital stay.

**Conclusions:**

Both UBE and PTED have favorable outcomes in patients with single-level LDH. However, PTED is superior to UBE in terms of short-term postoperative back pain relief and perioperative quality of life.

## Introduction

Lumbar disc herniation (LDH), with the disc material extruded outside the normal intervertebral space, is the main cause of low back and lower extremity pain (1). Although conservative care remains the main strategy for treatment, discectomy is required when clinical symptoms cannot be resolved *via* nonsurgical treatment (2, 3).

With advances in medical technology, open discectomy has been gradually replaced by minimally invasive spine surgery, and microdiscectomy has become an important part of the treatment of LDH (4). Facilitated by the development of endoscopic equipment and techniques, a variety of modified minimally invasive lumbar surgical techniques have been developed (5).

To protect the normal spinal structure, percutaneous transforaminal endoscopic discectomy (PTED) for LDH was developed after it was proposed by Yeung in 1997 and Hoogland in 2003 (6, 7). Based on the safety area of the lumbar posterolateral zone, PTED could remove the herniated disc effectively under local anesthesia (8). With favorable clinical results and good perioperative quality of life, PTED is appreciated by many spinal surgeons and patients (9). However, in addition to its steep learning curve, this technique requires specialized equipment, and discectomy is limited by the working channel (10).

In recent years, unilateral biportal endoscopic discectomy (UBE) with an arthroscopy system has become increasingly popular, especially in Asia (11). UBE decompression is performed on the ipsilateral side *via* two small separated surgical portals. Compared to PTED, UBE is not limited by the uniportal tube (12). The surgeons could perform discectomy and annulus fibrosus suture in a magnified surgical field with a high-definition arthroscope and a clear surgical field with saline irrigation (13). Previous reports have also shown satisfactory clinical outcomes of UBE for cervical and thoracic spinal disease (14, 15).

Few studies have directly compared PTED and UBE for the treatment of LDH (16). Therefore, to explore the differences between the two surgical techniques, this study compared the clinical efficacy of UBE and PTED for treating single level LDH.

## Methods

### Demographic characteristics

We performed a retrospective review in two hospitals of patients who underwent UBE and PTED from January 2020 to November 2021 after a diagnosis of single-level LDH. These surgeries were performed by two experienced surgeons. They had open lumbar surgery experience of more than 15 years, and PTED and UBE experience of more than 3 years. The baseline parameters of their demographic characteristics are given in Table 1. This retrospective study was approved by the Ethics Committee of the Chengde Medical University Affiliated Hospital, and written informed consent was obtained from the participants before data collection. The inclusion criteria were: (1) significant lower extremity radiating pain due to single-level LDH on x-ray, CT and MRI; (2) the absence of improvement after conservative treatment for at least three months; and (3) follow-up of at least 12 months after surgery. The exclusion criteria were: (1) mainly back pain symptoms or segmental instability on x-ray; (2) prior lumbar surgery; (3) tumor, infection, or trauma; and (4) inability to tolerate general anesthesia. The perioperative outcomes and complications were reviewed. An independent surgeon evaluated the VAS and ODI scores and modified MacNab criteria. x-ray examinations were performed one year after surgery to assess the segmental instability in both groups.

**Table 1:** Preoperative demographic characteristics.

Characteristics	UBE group (*n* = 30)	PTED group (*n* = 32)	*P* value
Age (years)	46.70 ± 11.62	48.03 ± 13.20	0.676
Sex (male/female)	11/19	13/19	0.749
Duration of symptoms (month)	13.53 ± 9.00	12.90 ± 9.17	0.787
Comorbidities (yes/no)	12/18	15/17	0.585
Side (right/left)	14/16	15/17	0.987
Level (L4-L5/L5-S1)	17/13	17/15	0.779
Type of disk herniation			0.769
Protrusion	10	8	
Sequestered	16	19	
Migration	4	5	

### Surgical procedures

**Figure 1:**
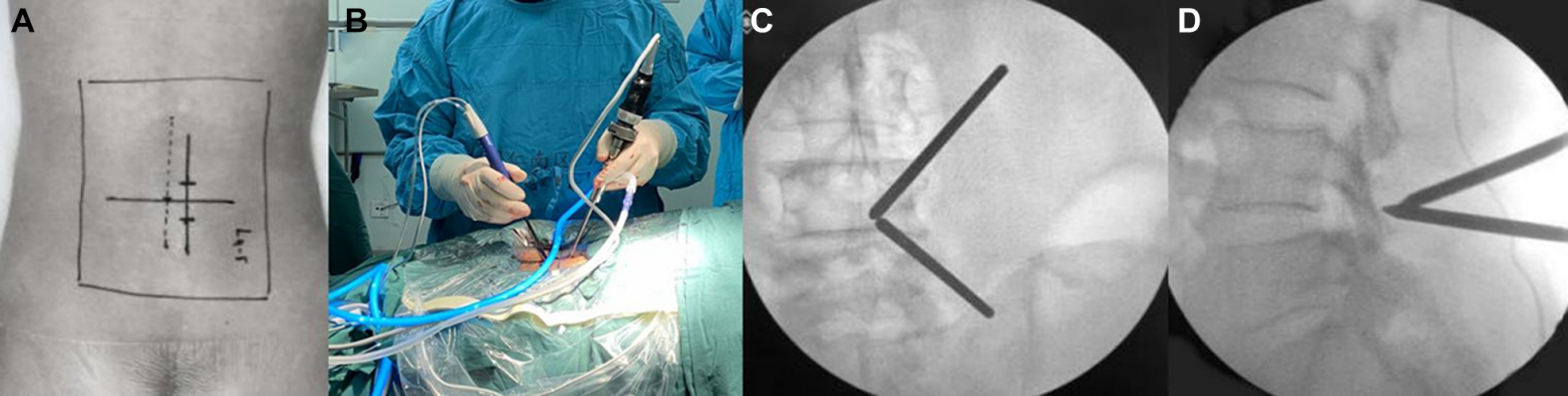
Intraoperative positioning and access establishment of UBE. (**A,B**) Body markers of L4/5 intervertebral space and the surgical approach. (**C,D**) The frontal and lateral view of the viewing and working portal.

**Figure 2:**
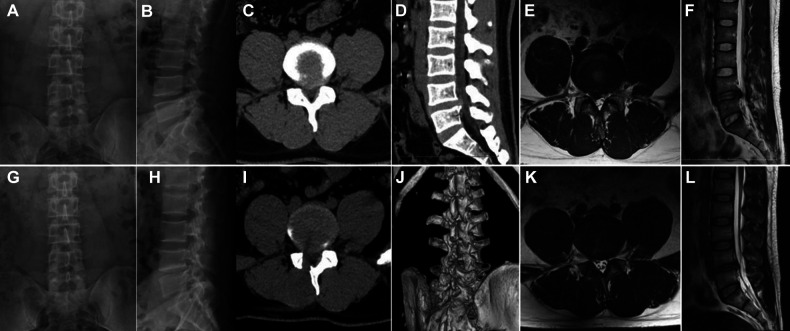
Pre- and postoperative x-ray, CT and MRI of UBE. (**A,B**) Preoperative x-ray. (**C–F**) Preoperative CT and MRI showing disc herniation. (**G,H**) Postoperative x-ray. (**H,I**) Postoperative CT and MRI showing the extruded disc was removed.

For the UBE group, the surgical procedure (based on the L4-L5 segment of LDH) was performed following methods reported in the literature (17). After successful general anesthesia with tracheal intubation, the patient was placed in a prone position with the abdomen draped, and the L4-L5 intervertebral space was marked with x-ray fluoroscopy. The initial target point is located at the junction of the inferior lamina and the spinous process of L4. The surgical bed is adjusted until the responsible intervertebral space is vertical to the floor to make the first horizontal line, and the second line is drawn along the inner edge of the pedicles of L4-L5. The observation and operation incision points on the body surface along the second line were approximately 0.5–1.0 cm from the intersection of the two lines (Figure 1). Two incisions were made, 0.8 cm–1.0 cm long, in the skin and subcutaneous fascia. Then, we bluntly expanded and separated the soft tissue covering the surface of the lamina to form the working and observation portals. With irrigation, the arthroscopic system was inserted into the observation portal. The soft tissue on the surface of the intervertebral space was removed by the plasma scalpel in the working portal. Next, the ipsilateral spinolaminar junction at the L4-L5 level was identified, laminotomy was performed with part of the inferior lamina of L4, and the superior lamina of L5 was removed with a drill. After the exposed ligamentum flavum was removed, the discectomy was conducted with Kerrison forceps. Finally, a drainage tube was placed after hemostasis. x-ray, CT and MRI were performed after surgery (Figure 2).

**Figure 3:**
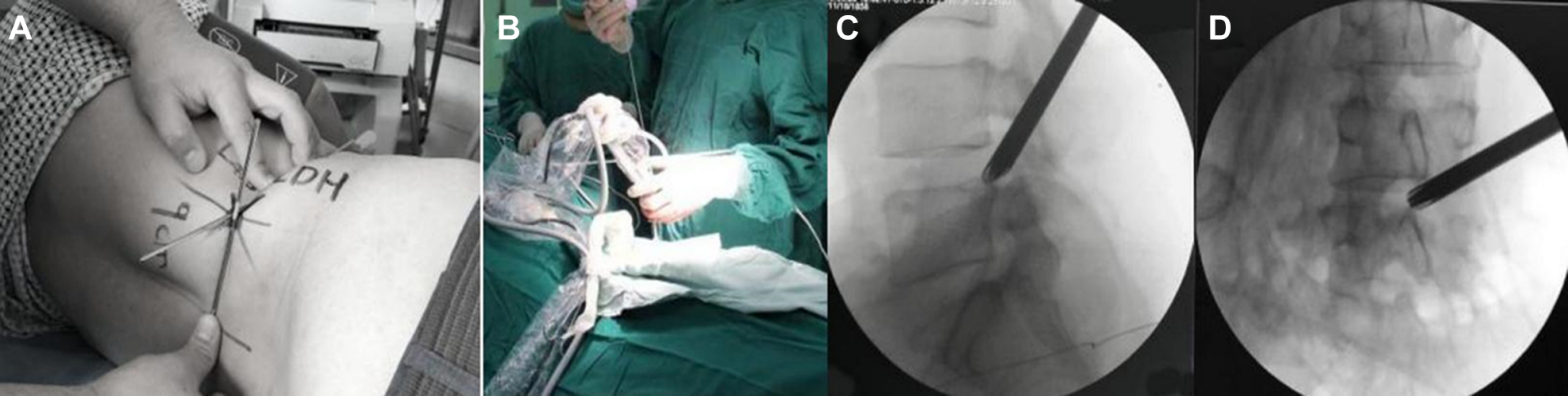
Intraoperative position and access establishment of PTED. (**A,B**) Body marker of L4/L5 intervertebral space and the surgical approach. (**C,D**) The lateral and frontal view of the working cannula.

**Figure 4:**
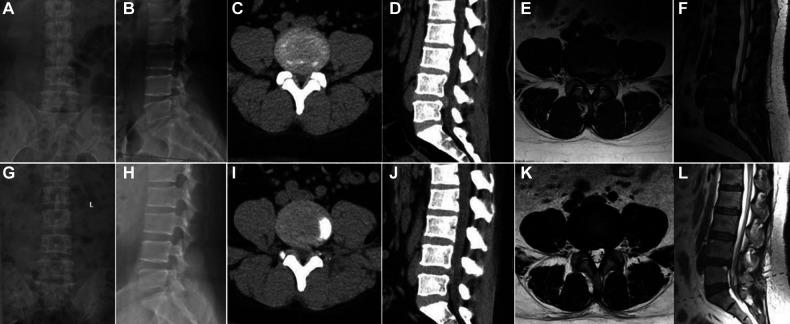
Pre- and postoperative x-ray, CT and MRI of PTED. (**A,B**) Preoperative x-ray. (**C–F**) Preoperative CT and MRI showing disc herniation. (**G,H**) Postoperative x-ray. (**H,I**) Postoperative CT and MRI showing the disc was removed.

For the PTED group, the following steps (based on the L4-L5 segment of LDH) were performed following methods reported in the literature that we have published (18). A soft pillow was placed under the patient's waist while the patient was in the lateral decubitus position with their knee and hip flexed. The incision was located 8 cm–12 cm from the midline horizontally and 2 cm–4 cm above the iliac on the side with leg pain. A mixed local anesthetic, which consisted of 30 ml 1:200,000 epinephrine and 20 ml 2% lidocaine, was used. After 5 ml of the mixed anesthetic was inserted into the skin at the entry point, 20 ml was inserted into the trajectory, 15 ml was inserted into the articular process, and 10 ml was inserted into the foramen. Then, 0.8 cm–1.0 cm of skin and the subcutaneous fascia were incised. Drills were used to resect the ventral osteophytes on the superior articular process of L5. The PTED system (Hoogland Spine Products, Germany) was inserted (Figure 3). Parts of the ipsilateral ligamentum flavum and the extruded lumbar disc were completely resected with endoscopic forceps. The drainage tube was placed after hemostasis. X-ray, CT and MRI were performed after surgery (Figure 4).

### Statistical analysis

The SPSS 26 program (IBM Corporation, United States) was used for statistical analysis. Repeated-measures analysis of variance was used to compare the VAS and ODI scores between the two groups. The independent-sample t test and Mann–Whitney *U* test or Fisher's exact test were used to assess the demographic characteristics and the perioperative outcomes. The level of statistical significance was set at *P* < 0.05.

## Results

### Perioperative outcomes

Of the 62 patients who met the study inclusion criteria, 30 underwent UBE, and 32 underwent PTED. The surgical parameters, including the operative time, estimated blood loss, incision length, times of x-ray, length of hospital stay and number of complications, are shown in Table 2. Except times of x-ray, the perioperative outcomes of the patients who underwent PTED were better than those of the patients who underwent UBE.

**Table 2:** Perioperative outcomes.

Characteristics	UBE group (*n* = 30)	PTED group (*n* = 32)	*P* value
Duration of surgery (min)	84.17 ± 17.62	64.06 ± 14.73	0.00
Estimated blood loss (ml)	51.33 ± 18.33	13.13 ± 3.76	0.00
Incision length (cm)	2.27 ± 0.39	1.23 ± 0.25	0.00
Times of x-ray	6.13 ± 1.28	11.16 ± 3.71	0.00
Postoperative hospital stay (day)	4.83 ± 1.86	3.28 ± 1.08	0.00
Complications (yes/no)	3/27	2/30	0.884

### Clinical results

**Figure 5:**
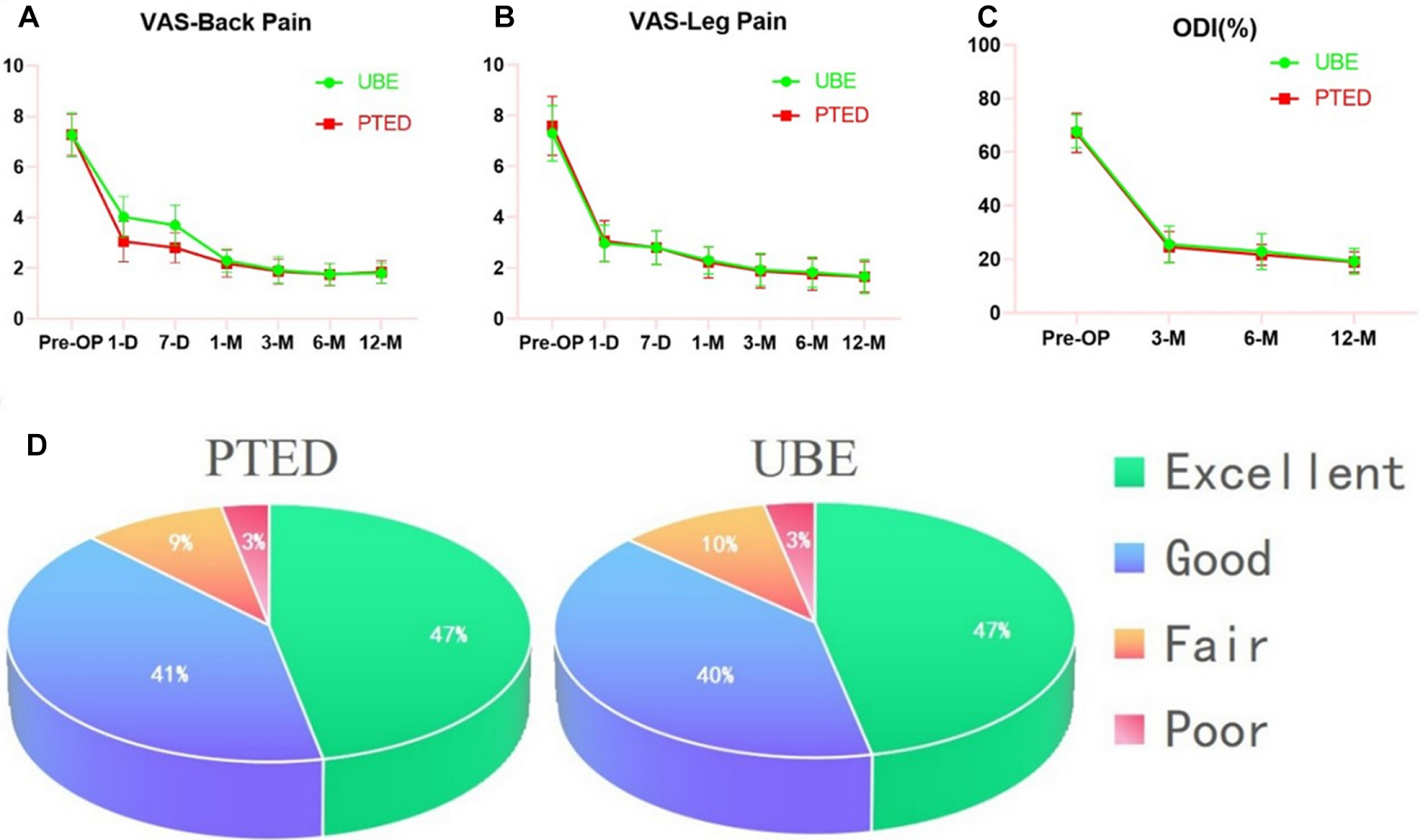
Clinical outcomes at different follow-up time points. (**A**) VAS score for back pain in both groups. (**B**) VAS score for leg pain in both groups. (**C**) ODI score for both groups. (**D**) The modified MacNab for both groups.

Preoperatively, the mean VAS and ODI scores were similar between the two groups. Compared to the UBE group, the PTED group had better VAS scores for back pain at 1 day and 7 days after surgery (3.06 ± 0.80 vs. 4.03 ± 0.81, *P* < 0.05; 2.81 ± 0.60 vs. 3.70 ± 0.79, *P* < 0.05). At 12 months, we observed similar improvements in the mean VAS scores for back and leg pain and ODI scores in the PTED and UBE groups (Figure 5). Moreover, there were no differences between the groups at any follow-up time point after the first month (*P* > 0.05). Based on the modified MacNab criteria, the good-to-excellent rate was 86.7% (26/30) in the UBE group and 87.5% (28/32) in the PTED group at the final follow-up. During the one-year follow-up in both groups, no segmental instability occurred.

### Complications

Three patients in the UBE group had a dural tear, and one experienced cerebrospinal fluid leakage and headache after the operation. These symptoms were relieved by adequate rest in the hospital bed and prolonging the drainage time. In the PTED group, one patient complained of dysesthesia and weakness of the tibialis anterior, which improved after a week with neurotrophic drugs; another patient had a dural tear without cerebrospinal fluid leakage. There were no serious complications related to surgery.

## Discussion

The significant improvements in the VAS score, ODI score and modified MacNab criteria revealed acceptable patient satisfaction in both groups, indicating that both PTED and UBE were effective in treating LDH. However, apartfrom times of x-ray, PTED is advantageous regarding the operative time, estimated blood loss, incision length, length of postoperative hospital stay, and short-term postoperative back pain relief.

For the surgical treatment of LDH, the most classic decompression is open laminectomy with or without fusion (19). However, open laminectomy destroys the paraspinal muscles and the posterior stabilizing structures. Therefore, a less invasive approach is needed to reduce injury and minimize surgical wounds during the treatment of lumbar disease (20).

As a microinvasive technique, PTED is widely applied for treating LDH with faster postoperative rehabilitation and less surgical injury. Compared to conventional open discectomy, PTED has the advantage of protecting the posterior ligament structures, facet joint and lamina. It avoids the need for nerve-root retraction and has a shorter hospitalization, reduced intraoperative bleeding, and faster recovery (21).

PTED can be completed under local anesthesia (22). After lidocaine combined with epinephrine hydrochloride solution is administered, the surgical field is clearer without obvious drug-related complications. The pressure of irrigation can also be appropriately reduced, theoretically reducing the incidence of spinal hypertension reactions (23). In addition, if the surgical equipment stimulates the nerve root during the operation, the awake patient will experience an abnormal sensation, and the surgeon can stop the process in a timely manner. The patient can be asked whether they subjectively feel their symptoms being alleviated, and the straight-leg test can be performed; these responses can be used to determine whether the operation should be terminated. Local anesthesia also reduces complications related to general anesthesia in elderly patients.

However, most hospitals in developing countries cannot afford to purchase these types of equipment and cannot master the technology quickly due to its steep learning curve. In addition, it is not easy to place the tube at the target point of the lateral approach if the iliac crest is high. In addition, the working places and visual field are limited to a single rigid working cannula.

Since first reported by De Antoni in 1996, UBE with arthroscopy has achieved good clinical effects (24). However, the development of UBE was limited due to the lack of power motor drills and the radiofrequency used to remove the lamina and achieve hemostasis. In recent years, with the emergence of endoscopic surgical instruments, UBE has been widely used in the treatment of LDH and lumbar spinal stenosis (25, 26).

Soliman proposed the application of this minimally invasive technology for the treatment of LDH in 2013 (27). He concluded that the surgical field of vision was expanded with different channels, and vascular bleeding was less under irrigation. The decompression process and instruments of UBE are similar to those used for open posterior discectomy, and thus this procedure can be carried out after only a short training period (28). Therefore, the learning curve of UBE is relatively flat and short. Xu demonstrated that the learning curve for mastering UBE is 54 cases (29).

The operating instruments and observation port are in different channels. The working port does not restrict the operating instruments of UBE. The working efficiency can be greatly improved with the use of conventional, large-sized surgical instruments, such as an osteotome, rongeur, forceps, and nerve retractor (30). In addition, surgeons in developing countries can complete the procedure without purchasing specialized supporting surgical instruments and other endoscopic systems. Moreover, unlike PTED, the UBE approach is not affected by a high iliac crest (31).

In our research, the operative time of UBE is longer than PTED. For one reason, the operative time for UBE is from the beginning of general anesthesia until a drainage tube is placed after hemostasis; the operative time for PTED is from the insertion of a local anesthetic to a drainage tube placed. For another, before laminotomy, the water pressure is 35 cm–40 cm H2O (32). But when performing the discectomy, to avoid potential neurological complications caused by the increased epidural and intracranial pressure and muscle edema caused by the high pressure of irrigation fluid, we lower the water pressure to 25 cm H2O (33). The time of hemostasis may be longer. So, the total operative time of UBE is longer than PTED in our research. But this does not mean that the efficiency of UBE is inferior to PTED in the progress of discectomy.

As for times of x-ray, the UBE group is superior to PTED in this research(6.13 ± 1.28 vs. 11.16 ± 3.71). Among the procedure of PTED, the times of x-ray was higher and mainly included: the process of local anesthesia, sequential dilators and bone drills insertion to expand the soft and osseous tissues by resecting the ventral osteophytes on the superior articular process, and the working cannula placement. In the UBE, the purpose of fluoroscopy is to find the junction of the inferior lamina and the spinous process and prevent mismaking of the target lumbar segment. So in terms of times of x-ray, the UBE group is superior to PTED.

However, the trauma of UBE is relatively larger than that of PTED (34). Due to the lack of a rigid cannula to dilate the soft tissue, the longissimus pectoralis and multifidus muscle need to be bluntly dissected to create a working space before decompression. The artificial creation of the operation spaces may damage the muscle attached to the lamina and the other anatomical structures. Therefore, theoretically, UBE would result in greater blood loss and worse postoperative back pain than PTED. The probability of cerebrospinal fluid leakage caused by dural injury when retraction of the nerve root is relatively high under general anesthesia (35).

In this research, three patients in the UBE group underwent dural tears when the anatomical structure was retracted to expose the disc. One of them experienced cerebrospinal fluid leakage and postoperative headache. The first dural tear occurred during the removal of the ligament flavum by the forceps with the low water pressure and the bleeding vision. The othe two dural tears were caused when the traversing roots was pushed by the assistant in a medial direction to expose the disc. We suggest that vigorous force cannot be used while pulling on the dura and an experienced assistant is needed. Besides, thorough hemostasis is needed when bleeding occurs before the next steps.

Additionally, one patient complained of weakness of the tibialis anterior in the PTED group. The working channel compresses the nerve root when the bone drill graves the upper articular of L5, which results in radicular symptoms. Another patient had a dural tear during the procedure but without cerebrospinal fluid leakage after the surgery. To avoid these complications, the surgeon should be careful when performing the foraminoplasty with a bone drill.

This study has some limitations. First, it was a retrospective study with a relatively short follow-up period and a small sample size. Second, the operation choices were limited. To confirm the long-term outcomes, a prospective and multicenter study with different surgical procedures and a larger sample size is necessary in future research.

## Conclusion

Both UBE and PTED showed favorable outcomes for the treatment of single-level LDH. With less bone and muscle damage, PTED under local anesthesia exhibited less intraoperative blood loss, a shorter operation time, and shorter postoperative hospitalization than the UBE group.

## Data Availability

The original contributions presented in the study are included in the article/Supplementary Material, further inquiries can be directed to the corresponding author.
